# A Flexible Ionically Conductive Biopolymer Hydrogel Interface for Physiological Signal Acquisition: A Chitosan–Glycerol–PVA Composite

**DOI:** 10.3390/ma19142973

**Published:** 2026-07-10

**Authors:** María Claudia Rivas Ebner, Giyeon Yu, Emmanuel Ackah, Seong-Wan Kim, Young-Seek Seok, Seung Ho Choi

**Affiliations:** 1 Department of Biomedical Engineering, Yonsei University, Wonju 26493, Republic of Korea; crivasebner@yonsei.ac.kr (M.C.R.E.); dbrldus074@yonsei.ac.kr (G.Y.); eackah.3@yonsei.ac.kr (E.A.); 2 Department of Agricultural Biology, National Institute of Agricultural Sciences, Rural Development Administration, Wanju 55365, Republic of Korea; tarupa@korea.kr; 3 Gangwon-do Agricultural Product Registered Seed Station, Chuncheon 24410, Republic of Korea; 4 Department of Integrative Medicine, Major in Digital Healthcare, Yonsei University College of Medicine, Seoul 06229, Republic of Korea

**Keywords:** hydrogels, chitosan, chitosan hydrogel, ECG, EMG, physiological signals

## Abstract

This study presents the development of a proof of concept, functional hydrogel interface designed for the acquisition of physiological signals, such as electrocardiogram (ECG) and electromyography (EMG). The hydrogel is synthesized using chitosan extracted from the shells of *Tenebrio molitor* larvae through a sustainable acid–alkaline protocol, blended with glycerol, polyvinyl alcohol (PVA), and ionized with NaCl to enhance conductivity. The resulting hydrogel membranes were cast and cut into circular shapes to provide a uniform contact geometry. The fabrication process yielded flexible membranes exhibiting ionic conductivity and partial surface conformity and handling stability. The extracted chitosan was characterized by Fourier-transform infrared spectroscopy (FTIR), degree of deacetylation (DDA), and molecular weight determination. Mechanical characterization included compression and tensile testing, while electrical characterization was performed through impedance spectroscopy and comparison with a commercial hydrogel interface. Functional evaluation was conducted through ECG and EMG signal acquisition under controlled experimental conditions. Preliminary in situ ECG and EMG recordings demonstrated successful signal acquisition using the proposed hydrogel interface. Future work may further investigate the mechanical and electrical behavior of the hydrogel under broader experimental conditions, as well as the optimization of the hydrogel formulation and extended physiological signal acquisition. Studies may help further characterize its potential as a chitosan-based bio interface material for bioelectrical sensing applications.

## 1. Introduction

With the advancement of technologies, particularly in soft interfaces and their demands for progress, multiple challenges remain in integrating characteristics such as reliability, low cost, and user-friendliness. In recent years, a wide range of sensors have been developed, with one of their main functions being the capture of electrophysiological signals, including electromyography (EMG), electrocardiography (ECG), electroencephalography (EEG), and electrooculography (EOG) [1].

To meet these requirements, new soft materials have been explored for the fabrication of interfaces. Among them, conductive hydrogels incorporating nanocomposites are considered promising candidates for bioelectronics, as their tunable mechanical and electrical properties allow for optimization of bio signal monitoring [2]. Hydrogels are three-dimensional (3D) polymeric networks that contain large amounts of water. Due to their high water content, porosity, softness, and 3D organization, they resemble the natural microenvironment of living tissues [3]. Owing to these properties, hydrogels have become an important element in biomedical science, with applications ranging from tissue regeneration and drug delivery to medical implants [4].

However, the search for environmentally friendly polymers that meet the requirements of biocompatibility, biodegradability, and low cost continues to be a major challenge. Hybrid hydrogels, composed of synthetic polymers combined with natural-source polymers, represent a viable option. They integrate the advantages of both types of polymers: the biocompatibility and bioactivity of natural polymers with the adjustable mechanical strength and stability of synthetic ones. In this way, hybrid hydrogels overcome the limitations of traditional hydrogels, especially when a single polymer cannot satisfy mechanical, chemical, and biological requirements [4].

In this context, chitosan (CS) stands out as a versatile natural polymer. CS is a polysaccharide mainly derived from the exoskeletons of crustaceans and insects, and it has attracted significant attention for its remarkable properties and potential biomedical applications [5]. In this work, CS was extracted from *Tenebrio molitor* (mealworm) shells. Structurally, CS is a linear copolymer composed of randomly distributed β-(1-4)-linked D-glucosamine (deacetylated units) and N-acetyl glucosamine (acetylated units). It is the second most abundant natural polysaccharide after cellulose, obtained by alkaline deacetylation of chitin, which is the main structural component of arthropod exoskeletons [6].

Insects represent a plentiful and sustainable source of chitin and chitosan, offering unique advantages over crustaceans. They reproduce rapidly, have short life cycles, and require minimal resources for rearing, ensuring a steady and accessible supply. Moreover, using insects as raw material reduces the environmental footprint compared to crustacean-derived sources, which aligns with the growing demand for eco-friendly alternatives [7]. From a biomedical perspective, insect-derived CS also shows the advantage of reduced allergenicity compared to crustacean-derived CS, where allergic reactions are mainly associated with proteins such as tropomyosin [7]. Another highly relevant property of CS is its antibacterial activity. For example, T. Molitor chitosan showed inhibition zones of 1–2 mm against Bacillus cereus, Listeria monocytogenes, Escherichia coli, Pseudomonas aeruginosa, and Staphylococcus aureus, all of which are bacteria commonly found in hospital environments and associated with nosocomial infections [8,9,10,11].

The main disadvantage of CS is its fragility, which can be improved by mixing it with flexible synthetic polymers such as polyvinyl alcohol (PVA). CS contains hydroxyl and amine groups that interact strongly with PVA, forming hydrogen bonds that release energy (negative enthalpy of mixing). PVA-based hydrogels also exhibit flexibility, stretchability, and resistance to fatigue, and their porous structure provides natural channels for rapid ion transport, which makes them well suited for applications such as electronic skin [12].

To address the inherent limitations of physically weak polymer networks, various crosslinking strategies have been explored in recent years. Among them, physical crosslinking of chitosan–PVA hydrogels prepared via freeze–thaw techniques has been extensively investigated due to their accessibility and relative ease to achieve and ease of fabrication. Hydrogels of this type have been used mostly for drug delivery, structural stability studies and basic mechanical analysis, often focusing on physicochemical characterization and mechanical behavior [13]. However, comparatively fewer studies have addressed the integrated mechanical and electrical response of CS–PVA hydrogels in the context of sensing applications [14].

In most reported chitosan–PVA hydrogel composite studies, commercially available chitosan is employed as the main polymer source, with limited discussion regarding the polymer origin or extraction route, and the focus typically lies on physicochemical characterization or basic mechanical performance [13,14,15,16]. In contrast, the present work utilizes chitosan extracted in-house from an insect-derived source. No direct comparison between chitosan sources is intended.

In this work, we focus on the development and proof-of-concept functional evaluation of a physically crosslinked chitosan composite hydrogel prepared via a freeze–thaw technique. The hydrogel was mechanically and electrically characterized and evaluated using physiological signals (ECG and EMG) as functional benchmarks through integration with a commercial Ag/AgCl electrode platform. This study examines the behavior of a physically crosslinked biopolymer network as a conductive interface material for physiological signal acquisition.

## 2. Materials and Experimental Methods

### 2.1. Materials

All materials used in this study are listed below. Mealworm (*Tenebrio molitor*) shells were obtained as biowaste from the Gangwon-do Agricultural Product Registered Seed Station (Chuncheon, Republic of Korea). Chitosan was extracted from the mealworm shells in our laboratory and prepared as a 4% (*w*/*v*) solution.

Sodium chloride (NaCl, 99.5%, special grade; SAMCHUN Chemical Co., Ltd., Gangnam-gu, Seoul, Republic of Korea), glycerol (DAEJUN Chemicals & Metals Co., Ltd., Gyeonggi-do, Pyeongtaek, Republic of Korea), and polyvinyl alcohol (PVA; SAMCHUN Chemical Co., Ltd., Gangnma-gu, Seoul, Republic of Korea) were used during the hydrogel preparation. Crystal-grade polyester Petri dishes (35 × 10 mm; SPL Life Sciences Co., Ltd., Pocheon-si, Gyeongii-do, Republic of Korea) were used as casting mold for film formation.

Fifteen-milliliter centrifuge tubes (Nest Biotechnology Co., Ltd., Wuxi, China) and 50 mL glass beakers (SIMAX, Sázava, Czech Republic) were used during material preparation and handling. Heating was performed using a hot plate (MSH-20D; Daihan Scientific, Wonju, Gangwon-do, Republic of Korea), and mass measurements were obtained using an analytical balance (KERN ADB; KERN & Sohn GmbH, Balingen, Germany).

Electrical measurements and data acquisition were performed using a 2425 100 W SourceMeter, an Arduino Uno microcontroller board, and an integrated pulse oximeter and heart rate biosensor module (MAX30102, green LED). Circuit assembly and signal interfacing were carried out using a solderless breadboard. Commercial electrodes used as reference were disposable Ag/AgCl-monitoring electrodes (Kendall™ ARBO H124SG, Covidien/Medtronic), consisting of a polymer Ag/AgCl-sensing element integrated with a pre-gelled conductive hydrogel adhesive layer, designed for ECG/EMG monitoring applications and low interface impedance on the order of ~200 Ω under standard testing conditions (AAMI EC12). The same architecture of these commercial electrodes (Ag/AgCl sensing element and adhesive layer) was used to evaluate and test the chitosan hydrogel.

### 2.2. Chitosan Extraction Procedure

Chitosan was obtained from *Tenebrio molitor* larvae shells through a multistep extraction process developed for this work based on previously reported protocols for insect-derived chitin and chitosan, combined with experimental optimization [17,18,19,20,21].

The extraction workflow was guided by literature recommendations regarding reagent type, concentration ranges, temperature and processing time, while specific conditions were iteratively adjusted based on experimental observations obtained during the processing of *Tenebrio molitor* larvae biomass [18,22,23].

**Figure 1:**
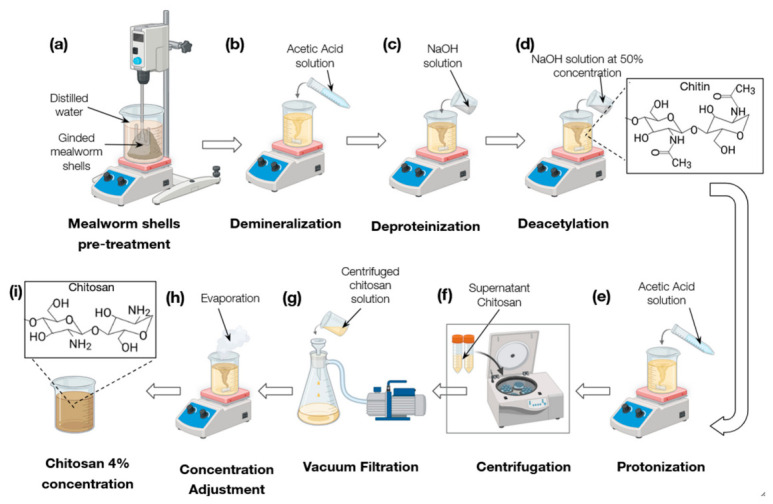
Step-by-step workflow for extracting chitosan from *Tenebrio molitor* larvae shells and preparation of a 4% chitosan solution. (**a**) Mealworm shells are ground and hydrated as initial pre-treatment. (**b**) Acetic acid is added to initiate the demineralization step. (**c**) NaOH solution is incorporated for deproteinization. (**d**) A high-concentration NaOH treatment is applied to deacetylate chitin. (**e**) The resulting chitin is protonated using acetic acid. (**f**) The mixture is centrifugated to separate the supernatant containing solubilized chitosan. (**g**) The chitosan solution is filtered under vacuum to remove remaining particles. (**h**) Controlled evaporation is used to adjust the final solution concentration. (**i**) Final 4% chitosan solution obtained after processing.

As illustrated in Figure 1, the process consisted of raw shell preparation, demineralization, deproteinization, alkaline deacetylation, protonation, and concentration adjustment. The selected conditions were applied consistently throughout this study to obtain a chitosan solution suitable for subsequent physically crosslinked hydrogel fabrication and functional testing.

#### 2.2.1. Preparation of the Raw Shells

A total of 25 gr of *Tenebrio molitor* larvae shells was weighed, ground using an electrical hand blender (PHILIPS Hand Blender 3000 series, HR2520/00, Amsterdam, The Netherlands), and transferred into a 1800 mL glass beaker. Although particle size was not formally measured, the material was visually confirmed to be coarse fine powder. The ground shells were then hydrated in 750 mL of distilled water and kept at 70 °C for 12 h under continuous stirring.

After hydration, the suspension was filtered using a standard laboratory strainer to separate the solid fractions. The shells were rinsed with cold distilled water, manually stirred, and filtered again. This washing step was repeated five times to ensure removal of residual soluble components. In this protocol, the same washing procedure is applied every major extraction step, except for the final filtration and concentration-adjustment step. This initial preparation step corresponds to Figure 1a,b.

#### 2.2.2. Demineralization

As illustrated in Figure 1c, demineralization was carried out through an acid-based decalcification step using acetic acid. This process removes inorganic compounds (mainly calcium carbonate) from the *Tenebrio molitor* larvae shells, facilitating the isolation of chitin and subsequently chitosan [24]. When acetic acid reacts with calcium carbonate, generating carbon dioxide and forming water-soluble calcium acetate, water and carbon dioxide are generated, effectively dissolving the mineral fractions of the shells [25]. Demineralization is essential for obtaining high-purity chitin, as residual minerals content can negatively affect the performance and consistency of the final biopolymer [26].

To prevent undesired depolymerization, both temperature and reaction time were selected. For this step, 35 mL of glacial acetic acid (≥99.7%, Daejung Chemicals & Metals Co., Ltd., Siheung, Republic of Korea) were diluted in 565 mL of distilled water to prepare a 5.8% *v*/*v* acetic acid solution (final volume of 600 mL). The hydrated shells were placed into a 1000 mL beaker, and the acid solution was added slowly to avoid localized over-reaction. The mixture was then covered and stirred at 70 °C for 12 h. After completion, the solids were collected by filtration and washed five times with distilled water, following the same washing protocol described in Section 2.2.1.

#### 2.2.3. Deproteinization

This step removes residual proteins and other organic components associated with the chitin matrix. In insect cuticles, chitin fibrils are organized within a protein-rich matrix, and protein chitin interactions contribute to the composite architecture [27]. Alkaline treatment with NaOH is widely used for deproteinization because it promotes protein removal from the biomass, improving chitin purification prior to deacetylation in chitosan [28]. Reported protocols commonly use NaOH in the molar range (≈1 M), often combined with elevated temperature (∼70–100 °C) and variable times depending on the insect source and process design [29].

In the context of this work, 50 g of sodium hydroxide (NaOH) pellets was gradually dissolved in 150 mL of distilled water under continuous stirring. The resulting solution was poured over the washed, semi-wet *Tenebrio molitor* larvae shells, which occupied 150–200 mL of volume, until reaching a final volume of 500 mL, yielding an effective NaOH concentration of approximately 3.6–4.2 M during deproteinization. The solution was magnetically stirred at 300–400 rpm, covered, and maintained at a temperature of 70 °C for 24 h. After the reaction was complete, the solid fractions were collected by filtration and thoroughly washed with distilled water until neutral pH.

#### 2.2.4. Deacetylation

The process of deacetylation converts chitin into chitosan by removing acetyl groups from N-acetyl-D-glucosamine units, thereby increasing the fraction of free amino groups (–NH_2_) [30]. This transformation is fundamental because the degree of deacetylation (DAA) strongly affects key physiochemical properties, including solubility/ionization behavior, cationic charge density, chemical reactivity, biodegradation behavior, and interactions with metal ions, which collectively influence suitability for biomedical and functional-material applications [31].

Among the available methods, deacetylation using concentrated sodium hydroxide remains the most applied approach in insect-derived chitin processing to obtain chitosan with effective DDA [32]. In this work, the chitin obtained after deproteinization was dispersed in 200 mL of a 50% (*w*/*v*) NaOH solution, prepared by dissolving 100 g of NaOH pellets in distilled water. The solution was placed in a 500 mL beaker, yielding a final reaction volume of 300 mL. The mixture was magnetically stirred at 300–400 rpm and heated to 120 °C for 24 h, in a covered beaker. Upon completion, the resulting chitosan was collected by filtration using standard laboratory filter paper and washed with distilled water.

#### 2.2.5. Protonation

Chitosan is generally insoluble in water and in many organic media, but it dissolves in aqueous acidic environments below approximately pH 6–6.5 due to protonation of the primary amine groups (CS–NH_2_ → CS–NH_3_^+^). This protonation increases electrostatic repulsion along the polymer chains, promoting chain solvation and expansion and thereby enabling dissolution. Acetic acid is commonly used to solubilize chitosan, and increasing acid concentration increases the degree of protonation and can improve dissolution completeness, which is relevant for preparing homogeneous casting solutions for film formation [33]. For this step, the chitosan extracted in the previous step was dissolved in a 4% (*v*/*v*) acetic acid solution. The acid solution was prepared by adding 6 mL of glacial acetic acid (≈99% purity) to 144 mL of distilled water, resulting in a total volume of 150 mL at approximately 4% (*v*/*v*). The semi-wet chitosan was placed in a clean 200–250 mL beaker, and the acetic acid solution gradually poured over it. The beaker was covered, and the solution was magnetically stirred at ~300–400 rpm and maintained at 50 °C for 24.

#### 2.2.6. Filtration

Following the previous step, the viscous chitosan mixture was subjected to centrifugation. The 150 mL suspension was evenly distributed into four 50 mL centrifuge tubes and processed at 4500× *g* for 10 min. The clear and viscous supernatant was poured into a clean 200–250 mL beaker, while the pellet containing residual organic matter was discarded. Because of the high viscosity of the solution, vacuum filtration was used to accelerate the process. The supernatant was filtered under pressure using a Büchner funnel and qualitative filter paper. The filtered material was collected in a clean 200–250 mL beaker and reserved for the subsequent processing step.

#### 2.2.7. Concentration Adjustment

The chitosan solution was prepared at 4% (*w*/*v*), a concentration commonly used in literature to obtain workable chitosan solutions for casting and biomaterial processing [34]. To determine its initial concentration, a 5 mL aliquot was transferred into a pre-weighed Petri dish and dried in an oven at 30 °C for 2~3 h. The mass of the dried chitosan was recorded, and the concentration (mg/mL) was calculated using:

$$\mathrm{Concentration}\;(w/v,\;\%)=\frac{\mathrm{Mass}\;\mathrm{of}\;\mathrm{solute}\;(\mathrm{mg})}{\mathrm{Volume}\;\mathrm{of}\;\mathrm{solution}\;(\mathrm{mL})}$$

After determining the initial concentration, the total solution volume was adjusted to reach the target concentration of 4%. This was accomplished by controlled evaporation at 90 °C, while continuously monitoring the solution to avoid overheating, degradation or over evaporation. The required final volume as well as the amount of solvent that needed to be evaporated, was calculating using the standard dilution equation:

$$C_{1}V_{1}=C_{2}V_{2}$$

### 2.3. Determination of Degree of Deacetylation and Molecular Weight

The degree of deacetylation (DDA) of the extracted chitosan was estimated using Fourier Transform Infrared Spectroscopy (FTIR). FTIR spectra were recorded to identify the characteristic functional groups of chitosan. The broad absorption band around 3410 cm^−1^ was attributed to O–H and N–H stretching vibrations, while the peaks at 2922 and 2877 cm^−1^ corresponded to C–H stretching vibrations. The amide I and amide II bands were observed near 1632 cm^−1^ and 1546 cm^−1^, respectively, associated with residual acetyl groups in the polymer structure.

The DDA was calculated based on the relative absorbance of the amide I band (A_1632_) and the hydroxyl band (A_3410_), according to the following expression:

$$\mathrm{DDA}(\%)\;=\;100\;-\left(\frac{A_{1632}}{A_{3410}}\;\times \;75\right)$$

where $A_{1632}$ and $A_{3410}$ correspond to the absorbance values of the amide I and hydroxyl bands, respectively.

The molecular weight of chitosan () was determined using static light scattering (SLS) based on the Debye equation. The relationship between scattering intensity and concentration is given by:

$$\frac{\mathrm{KC}}{R_{\theta }}=\left(\frac{1}{M_{w}}+2A_{2}C\right)\left(n\right)$$

where C is the concentration of the sample, $R_{\theta }$ is the Rayleigh ratio representing the ratio of scattered to incident light intensity, $M_{w}$ is the weight-average molecular weight, and $A_{2}$ is the second virial coefficient reflecting intermolecular interactions. The optical constant K is defined as follows:

(1) $$K=\frac{4\pi^{2}}{\lambda_{0}^{4}N_{A}}{\left(n_{0}\frac{\mathrm{dn}}{\mathrm{dC}}\right)}^{2}\left(n\right)$$

where $\lambda_{0}$ is the wavelength of incident light in vacuum, $N_{A}$ is Avogadro’s number, $n_{0}$ is the refractive index of the solvent, and dn/dC is the refractive index increment. The Rayleigh ratio $R_{\theta }$R was calculated using toluene as a reference standard:

$$R_{\theta }=\frac{I_{A}n_{0}^{2}}{I_{T}n_{T}^{2}}R_{T}\;\left(n\right)$$

where $I_{A}$ is the excess scattering intensity of the sample ($I_{\mathrm{sample}}$ − $I_{\mathrm{solvent}}$), $I_{T}$ is the scattering intensity of toluene, $n_{T}$ is the refractive index of toluene, and $R_{T}$ is the Rayleigh ratio of toluene.

In this study, 2% acetic acid was used as the solvent, and five concentrations in the range of 0.5–2.5 mg/mL were prepared and measured in accordance with the manufacturer’s guidelines. Scattering data were obtained using a Zetasizer Pro (Model: ZSU5800, Malvern Panalytical, Worcestershire, UK).

### 2.4. Hydrogel Fabrication

The fabrication parameters were selected based on reported protocols and commonly adopted ranges for physically crosslinked chitosan-based hydrogels, ensuring reproducible hydrogel formation and functional reliability, and all aqueous solutions used throughout the extraction and hydrogel fabrication processes were prepared using distilled water [35,36,37,38]. The hydrogel interfaces are intended for single-use, disposable application during short-duration physiological signal acquisition.

The hydrogel network was physically crosslinked using freeze–thaw cycles, a solvent-free approach that promotes the formation of PVA microcrystalline domains and increases physical entanglement/hydrogen bonding, thereby stabilizing the three-dimensional polymer network [39]. The quantities described below correspond to the fabrication of a single hydrogel sample with dimensions of 35 mm diameter and 0.30 mm thickness. First, 5 mL of 4% chitosan solution, 0.5 mL of 10% glycerol, and 0.1 g of NaCl were used. The 5 mL of chitosan was transferred into a 200 mL beaker and kept under continuous magnetic stirring at room temperature. The 0.5 mL of glycerol were slowly added, followed by the NaCl. The mixture was stirred for approximately 10–15 min.

**Figure 2:**
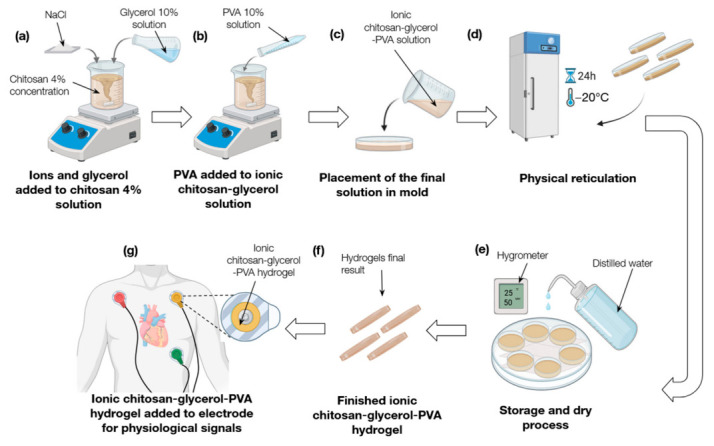
Step-by-step preparation of the ionic chitosan–glycerol–PVA hydrogel for biomedical electrode application. (**a**) NaCl and 10% glycerol are added to the previously prepared 4% chitosan solution. (**b**) A 10% PVA solution is then incorporated, and the solution is stirred for 30 min. (**c**) The resulting solution is poured into small Petri dish used as molds. (**d**) The molds undergo four cycles of physical crosslinking in a freezer. (**e**) After the crosslinking cycles, the hydrogels are dried under controlled humidity conditions (<50%) and cool temperature (~14 °C). (**f**) once dried, the hydrogels acquire a firm, gelatin-like appearance and consistency. (**g**) The hydrogel is removed from the mold and placed onto a commercial electrode Ag/AgCl metallic base without its hydrogel, to enable physiological signal acquisition.

After this initial step, 2 mL of 10% PVA solution was incorporated, and the resulting mixture was stirred for an additional 30–35 min to ensure complete homogenization (Figure 2b). Next, the final solution was poured into a small Petri dish serving as the casting mold (Figure 2c). The hydrogel was then subjected to physical crosslinking through freezing cycles. For this process, the molds were placed in a laboratory freezer at −20 °C for 24 h (Figure 2d). After each freezing cycle, the sample were thawed at a controlled cool temperature of approximately 14 °C (standard refrigerator conditions), while monitoring humidity to prevent excessive fast drying (Figure 2e).

The hydrogels underwent 4–5 freeze cycles to ensure consolidation of the polymer network. Once all cycles were completed and the hydrogels had fully thawed, they were removed from the Petri dish. Each hydrogel was cut into a circular shape using a cylindrical mold of 15.4 mm diameter and placed onto a commercial electrode, which the original hydrogel had been removed and replaced with our final chitosan-based hydrogel (Figure 2f). After the crosslinking cycles, the hydrogels are dried under controlled humidity conditions (<50%) and cool temperature (~14 °C) for 24 h. To minimize the presence of residual processing chemicals, the hydrogel was neutralized after fabrication using a NaOH solution to reverse the acidic conditions associated with chitosan protonation. Subsequently, the hydrogel was extensively washed with deionized water until a skin compatible (ph 4.5–6.5). Finally, the hydrogels were ready for physiological signal acquisition.

Prior to selecting the final hydrogel composition, several exploratory formulations were evaluated by modifying the glycerol concentration, PVA concentration, and NaCl content while maintaining a constant chitosan solution volume (5 mL). The purpose of this preliminary screening was to identify formulations capable of producing continuous self-supporting hydrogel films suitable for subsequent characterization. Representative formulations evaluated during this stage are summarized in Table 1.

### 2.5. Signal Acquisition Protocols

#### 2.5.1. Recording Protocol

ECG and EMG signals were collected for the purpose of comparing waveform morphology obtained using standard commercial Ag/AgCl electrodes with their original commercial hydrogel interface and the same electrodes after replacement of the hydrogel layer with the chitosan-based hydrogel developed in this study. The commercial electrode platform, including the original adhesive backing, electrode architecture, and geometric configuration, was otherwise preserved throughout all experiments, such that only the hydrogel interface material was modified. Recordings were obtained from healthy adult volunteers (*n* = 2) with no history of cardiovascular or musculoskeletal disorders that could influence the measurements. For both ECG and EMG measurements, the skin surface was cleaned and prepared using 70% isopropyl alcohol pads prior to electrode placement. In addition, electrode placement regions were marked on the skin using a surgical marker to improve positional reproducibility between recordings and no external pressure or force-control device was applied.

For ECG acquisition, each participant underwent two continuous 1 min recording in seated position at complete rest, one using commercial electrodes and the other, the modified electrode with chitosan-based hydrogel. For EMG acquisition was obtained from the tibialis anterior (TA) muscle activity. Each recording lasted 10 s, performed again under two conditions: one using commercial electrodes and one using the chitosan-based hydrogel electrodes. EMG was recorded from the right lower limb; participants were positioned sitting at rest, whit the knee slightly flexed and the heel with support, to allow for free ankle movement. In this position each participant was asked to perform three ankle dorsiflexion, one every two seconds, over the 10 s recording period.

#### 2.5.2. Electrocardiogram Signal Acquisition

After removing the commercial Ag/AgCl hydrogel from the commercial electrode metallic base, the chitosan-based hydrogel was cut into circular pads using a custom mold and mounted onto the metallic contact surface of the commercial electrode. Once placed in contact with the conductive pad, the modified electrode was ready for use. For ECG acquisition, three electrodes containing the chitosan-based hydrogel were positioned on the anterior thorax following the standard Lead II configuration, as recommended by the American Heart Association (AHA) and the Committee on ECG Standards. The placement relied on identifiable bony and muscular landmarks to ensure reproducibility and alignment with the cardiac electrical axis [40].

The RA electrode (right arm equivalent) was placed on the right infraclavicular region, into the 2dn intercostal space, immediately below the right clavicle and lateral to the sternum. The LA electrode (left-arm equivalent) was positioned symmetrically in relation to the RA electrode, on the left infraclavicular region. The RL electrode (ground) was placed in between the left lower thoracic region and the upper abdominal region, inferior to the costal margin and above the iliac crest. This configuration reproduces the vectors of a modified Lead II for continuous monitoring and is commonly used in clinical practice [41,42]. The ECG signal was recorded for 1 min per subject 40 times each one (*n* = 80). All electrodes were connected to the AD8232 Heart Monitor board, which interfaced with an Arduino UNO board for real time data collection and visualization in MATLAB R2024b.

#### 2.5.3. Electromyogram Signal Acquisition

Surface EMG recordings were obtained from the tibialis anterior (TA) using a standard bipolar configuration consistent with the SENIAM guidelines for non-invasive assessment of muscle activity [43]. The active and reference electrodes were positioned longitudinally along the line connecting the fibular head and the medial malleolus, at approximately one-third of this distance from the fibular head and the medial malleolus, at approximately one-third of distance from the fibular head, with the electrodes placed parallel to the muscle fibers as recommended for TA to maximize signal specificity and reduce crosstalk [44,45].

The inter-electrode distance was set to 20 mm center-to-center, following established EMG acquisition standards that optimize special sensitivity and minimize cross talking from adjacent muscles [46]. A ground electrode was placed over a distal bony prominence, specifically the lateral malleolus, to ensure low-impedance contact and mechanical stability, a placement commonly used in lower limb EMG studies [47]. EMG signals were recorded while the subject performed isolated ankle dorsiflexion in an open kinetic chain movement, with slight knee flexion, a protocol widely adopted to selectively activate the TA with minimal coactivation of synergist muscles [48].

### 2.6. Signal Processing

#### 2.6.1. ECG Signal Processing

ECG signals acquired with both commercial Ag/AgCl electrodes and the chitosan-based hydrogel electrodes were processed using an identical digital pipeline and were converted to millivolts using the hardware calibration factor (3.3/1023 × scale factor_. After conversion, raw signals were first corrected for DC offset using an adaptive exponential estimator (τ = 2.0 s) and were subsequently detrended using a cascade moving-median (0.60·Fs) and moving-average (0.20·Fs) baseline filter. The detrended signals were band-pass filtered using a 4th order zero-phase Butterworth filter (0.5–35 Hz). Powerline interference was removed using a 50 Hz IIR notch filter (Q = 40). For visualization, a Savitzky–Golay smoothing filter (2nd order, 7 points) was applied when available. Finally, signals were amplitude-normalized, using the 100th percentile absolute amplitude to allow for direct morphological comparison between electrode types.

#### 2.6.2. EMG Signal Processing

EMG recordings were converted to millivolts using the ADC calibration factor (0.00488 mV/count) and demeaned. Offline processing included 4th order zero-phase Butterworth band-pass filtering between 20–200 H, followed by attenuation of 60 Hz powerline interference using an IIR notch filter (r = 0.98). The cleaned signal was rectified and smoother with a 40 ms moving-average window to obtain the EMG envelope. Frequency-Doman analysis was performed using Welch’s method (500 sample window, 250 sample overlaps, 1024-point FFT), with PSD normalized to its maximum value and expressed in decibels.

### 2.7. Quantitative and Statistical Analysis

#### 2.7.1. ECG Spectral Analysis and Metrics

Power spectral density (PSD) was computed from filtered ECG signals in millivolts (sampling rate: 500 Hz) using Welch’s method (window length: 500 samples, overlap: 250 samples, NFFT: 1024). PSD was expressed in decibels as defined in Equation (1).

$${\mathrm{PSD}}_{\mathrm{dB}}(f)={10\log}_{10}\left(\mathrm{PSD}(f)\right)$$

Variability bands were computed as mean ± 1.96·SD across recordings at each frequency. The signal-to-noise ratio (SNR, dB) was calculated for each recording as follows:

$${\mathrm{SNR}}_{\mathrm{ECG}}(\mathrm{dB})\;=\;{20\log}_{10}\left(\frac{\mathrm{RMS}(x)}{\mathrm{RMS}({x\;-\;\mathrm{LP}}_{15\;\mathrm{Hz}}\left(x)\right)}\right)$$

where ${\mathrm{LP}}_{15\;\mathrm{Hz}}$ denotes a 15 Hz low-pass filter. R-peak amplitude was quantified by peak detection (MinPeakHeight = 0.5 mV; MinPeakDistance = 0.25 s) and defined as the maximum detected peak amplitude per recording. Quantitative metrics were computed from calibrated signals prior to any visualization-only normalization.

#### 2.7.2. EMG Spectral Analysis and Metrics

RMS amplitude was computed from raw EMG signals in millivolts (Fs = 1000 Hz). For each recording, rest RMS was calculated over the first 1 s segment. A 1 s contraction segment was selected by locating the maximum of a 1 s moving mean of the absolute raw signal, and RMS during contraction was computed over the corresponding 1 s window. The signal-to-noise ratio was defined as follows:

$${\mathrm{SNR}}_{\mathrm{EMG}}(\mathrm{dB})\;=\;{20\log}_{10}\left(\frac{{\mathrm{RMS}}_{\mathrm{cont}}}{{\mathrm{RMS}}_{\mathrm{rest}}}\right)$$

Power spectral density (PSD) was estimated from filtered EMG signals using Welch’s method (window length: 1000 samples, overlap: 500 samples, NFFT: 2000). PSD was averaged across recordings and plotted in dB; variability is reported as ±SD.

## 3. Results

**Figure 3:**
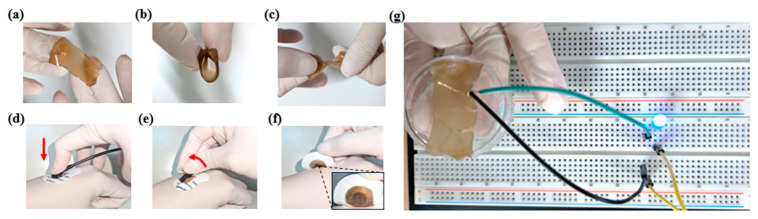
Qualitative assessment of the mechanical deformability, handling characteristics, and electrical continuity of the chitosan-based hydrogel prior to quantitative characterization. (**a**–**c**) Representative examples of manual deformation, including bending, folding, twisting, and stretching during handling, without visible fracture or structural failure. (**d**) Insertion of the lead wire connector pin into the electrode. The red arrow indicates the direction of the applied manual pressure during pin insertion. (**e**) Manual traction applied to the lead wire connector pin. The red arrow indicates the direction of the pulling motion used to remove the lead wire connector pin from the electrode. (**f**) Hydrogel after pin insertion and manual traction, showing the structural integrity of the hydrogel. (**g**) Demonstration of electrical continuity through the hydrogel using a simple circuit connected to a light-emitting diode (LED).

Prior to the electrical characterization, the chitosan-based hydrogel was qualitatively assessed in terms of its physical handling and practical integration with a commercial electrode substrate. After preparation, the hydrogel could be manually manipulated and cut to a rectangular shape. As shown in Figure 3a–c, the hydrogel could undergo bending, folding, twisting, and moderate manual stretching during handling without visible fracture or visual structural failure. The material maintained its integrity during handling and positioning, enabling direct electrical contact with the underlying Ag/AgCl metallic base.

Figure 3 also includes a demonstration in which a fragment of the chitosan-based hydrogel is incorporated into a simple electrical circuit composed of a power supply and a light-emitting diode (LED). Upon circuit connection, illumination of the LED is observed, indicating the presence of an electrically conductive pathway through the hydrogel.

### 3.1. Material Characterization

**Figure 4:**
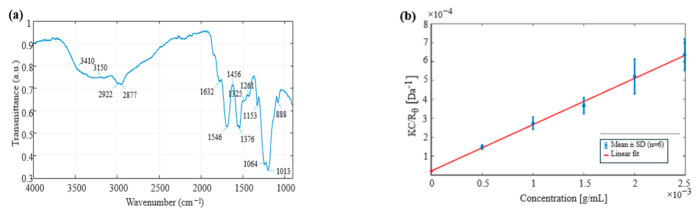
Physicochemical characterization of the extracted chitosan, where (**a**) the FTIR spectrum exhibits the characteristic absorption bands associated with chitosan functional groups, and (**b**) the Debye plot of chitosan dissolved in 2% acetic acid shows the linear regression used to estimate the weight-average molecular weight (M_w_ = 4.63 × 10^4^ g/mol), with data presented as mean ± standard deviation (*n* = 6) and a coefficient of determination of (R^2^ = 0.9954).

As shown in Figure 4a, the FTIR spectrum exhibited the characteristic absorption bands of chitosan [49]. A broad absorption band observed between 3410 and 3150 cm^−1^ was attributed to O–H and N–H stretching vibrations. The peaks at 2922 cm^−1^ and 2877 cm^−1^ were associated with symmetric and asymmetric C–H stretching vibrations. In the amide region, the bands observed at 1632 cm^−1^ and 1325 cm^−1^ were assigned to amide I and amide II vibrations, respectively, while the peak at 1546 cm^−1^ corresponded to N–H bending vibrations [49,50]. Additional absorption bands at 1456 cm^−1^ and 1376 cm^−1^ were associated with CH_2_ and CH_3_ bending vibrations, whereas the bands at 1261 cm^−1^, 1153 cm^−1^, 1064 cm^−1^, and 1013 cm^−1^ were attributed to O–H, C–O–C, and C–O vibrational modes [51]. The band observed at 888 cm^−1^ was associated with CH bending vibrations of the monosaccharide ring. Based on the FTIR analysis, the extracted chitosan exhibited a calculated degree of deacetylation (DDA) of 75.45%.

As shown in Figure 4b, linear regression analysis of the Debye plot yielded a slope of $2.4495\;\times \;10^{-1}$, an intercept of $2.1611\;\times \;10^{-5}\;{\mathrm{Da}}^{-1}$, and a coefficient of determination of $R^{2}=0.9954$. The weight-average molecular weight calculated from the intercept was $M_{w}\;=\;4.6273\;\times \;10^{4}\;g/\mathrm{mol}$. The Debye plot exhibited a linear relationship throughout the analyzed concentration range, indicating consistency in the scattering behavior of the polymer solution.

### 3.2. Mechanical Characterization

**Figure 5:**
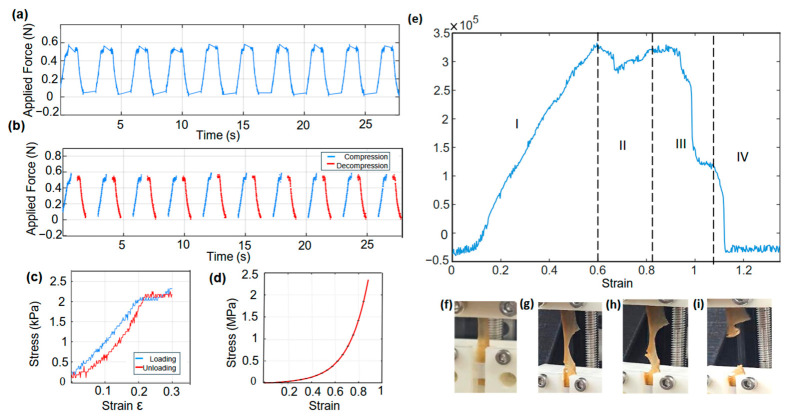
Mechanical characterization of the chitosan/PVA composite hydrogel. (**a**) Force–time response during cyclic compression. (**b**) Compression and decompression force values. (**c**) Stress–strain curve reconstructed from cyclic compression data. (**d**) Monotonic compressive stress–strain response. (**e**) Tensile stress–strain response until complete fracture, showing four stages (I–IV). Representative images of the specimen during tensile testing are shown for the intact condition (**f**), partial fracture (**g**), fracture progression (**h**), and the final stage prior to complete failure (**i**).

The mechanical response of the chitosan-based hydrogel was evaluated under cyclic compressive loading, and the resulting force–time and stress–strain behaviors are summarized in Figure 5. All cyclic compression measurements were performed by applying a maximum compressive deformation corresponding to 30% strain to a hydrogel film with an initial thickness of 0.3 mm. Figure 5a shows the force–time response recorded during repeated compression cycles at a fixed maximum compressive strain of 30%. A periodic force profile is observed over the measurement interval, with comparable force amplitudes maintained across successive cycles. The force signal increases during the compression phase and decreases during the subsequent release, reflecting the imposed cyclic deformation of the hydrogel film. Figure 5b presents the force data separated into compression and decompression phases. The compression data corresponds to the highest force values reached when the hydrogel is compressed to 30% strain, while the decompression data represents the force response measured during release from the same deformation level. This representation highlights the force values associated with the maximum resistance of the hydrogel during loading, as well as the residual resistance observed during the decompression phase within each cycle.

For stress calculation, the measured force was normalized using the known sample geometry, considering a rectangular contact area of 17.7 mm × 14.2 mm and an initial thickness of 0.3 mm. Based on this conversion, the stress–strain response reconstructed from the cyclic compression data up to 30% strain is shown in Figure 5c. The resulting loading and unloading curves exhibit a nonlinear compressive stress–strain behavior over the analyzed strain range, with stress increasing progressively as strain increased and distinct branches observed for the loading and unloading portions of the cycle. For reference, Figure 5d shows the compressive stress–strain response obtained under monotonic loading conditions, illustrating the stress evolution over a wider strain range and providing a complementary view of the material response under continuous compression.

The tensile stress–strain response of the chitosan hydrogel obtained during tensile testing is shown in Figure 5e. The recorded curve exhibited multiple load-drops and load-recovery events prior to complete fracture. Visual observations performed during testing revealed that these changes in the stress–strain response coincided with visible changes in the hydrogel film, as illustrated in Figure 5f,i.

Mechanical properties were determined from the tensile stress–strain curve. The Young’s modulus was obtained from the linear region identified within Phase I using linear regression (R^2^ = 0.9957), resulting in a value of 0.695 MPa. The hydrogel film reached an ultimate tensile strength (UTS) of 0.332 MPa at a strain of 0.5965. The elongation at break was 111.87%, and the calculated toughness, determined as the area under the stress–strain curve up to fracture, was 2.177 × 10^5^ J m^−3^.

To facilitate the description of the observed tensile behavior, the stress–strain curve was divided into four phases (I–IV). These phases were defined according to the features observed in the stress–strain curve and their correspondence with the visual appearance of the hydrogel film during the tensile test. Phase I corresponds to the initial portion of the stress–strain curve, where stress increased continuously with increasing strain while the hydrogel film remained visually intact (Figure 5f). Phase II begins after the first major reduction in stress. At this stage, a localized partial fracture was observed in the hydrogel film, while complete separation had not yet occurred (Figure 5g). Phase III corresponds to a region of the curve characterized by additional stress reductions. The corresponding image (Figure 5h) shows a larger fractured region than that observed in Phase II. Phase IV corresponds to the final portion of the stress–strain curve prior to complete fracture. At this stage, a limited connecting region of the hydrogel film remained visible (Figure 5i), followed by complete separation of the sample at the end of the test.

### 3.3. Electrical Characterization

**Figure 6:**
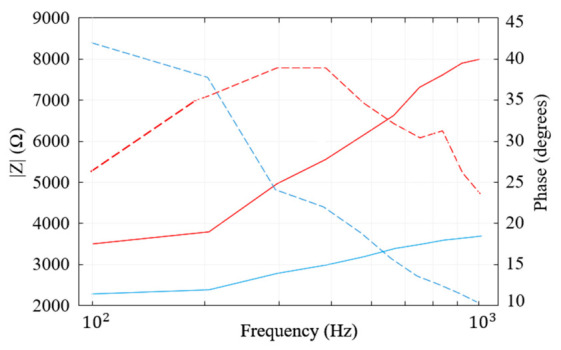
Impedance magnitude (|Z|) and phase response as a function of frequency are shown for the chitosan-based hydrogel and the commercial hydrogel, where solid lines correspond to |Z| and dashed lines represent phase, with red curves indicating the chitosan-based hydrogel, and blue curves indicating the commercial hydrogel.

The electrical characterization of the chitosan-based hydrogel was performed through impedance spectroscopy and compared with a commercial hydrogel interface (the hydrogel layer originally provided with the commercial Ag/AgCl electrode), as shown in Figure 6. The impedance magnitude (|Z|) and phase response were evaluated as a function of frequency. The impedance magnitude of both electrodes increased with frequency over the analyzed range, exhibiting comparable frequency-dependent trends. Specifically, the chitosan-based hydrogel showed a monotonic increase in |Z| from approximately 3.5 kΩ at 100 Hz to around 8.0 kΩ at 1 kHz, while the commercial electrode presented lower |Z| values, increasing from approximately 2.3 kΩ to 3.7 kΩ over the same frequency range. The phase response exhibited a frequency-dependent variation for both electrodes. The chitosan-based hydrogel displayed a higher phase value across most of the frequency range, with a maximum observed and intermediate frequencies followed by a gradual decrease at higher frequencies, whereas the commercial hydrogel showed a progressive decrease in phase with increasing frequency. Solid lines represent the impedance magnitude (|Z|), while dashed lines represent the phase response, with red curves indicating the chitosan-based hydrogel and the blue ones, the commercial hydrogel.

Using the measured impedance magnitude, a sample thickness of 0.03 mm, and an active electrode area of 50 mm^2^, the conductivity of the chitosan-based hydrogel was estimated to be on the order of 10^−6^ S cm^−1^. This estimation was calculated from the experimental geometry employed during impedance characterization and provides an approximate indication of the electrical transport behavior of the hydrogel under the conditions evaluated in this study.

The impedance values of the chitosan-based ionic hydrogel (3.5–8 kΩ) fall within the range reported for hydrogel-based and wet biopotential electrodes used in ECG and EMG applications, as established in classical comparisons of electrode types [52] and more recent hydrogel electrode reviews [53,54], where functional operation is maintained across the kΩ range without significant degradation in signal quality.

### 3.4. Physiological Signal Acquisition and Comparison

#### 3.4.1. ECG Signal Morphology Analysis

**Figure 7:**
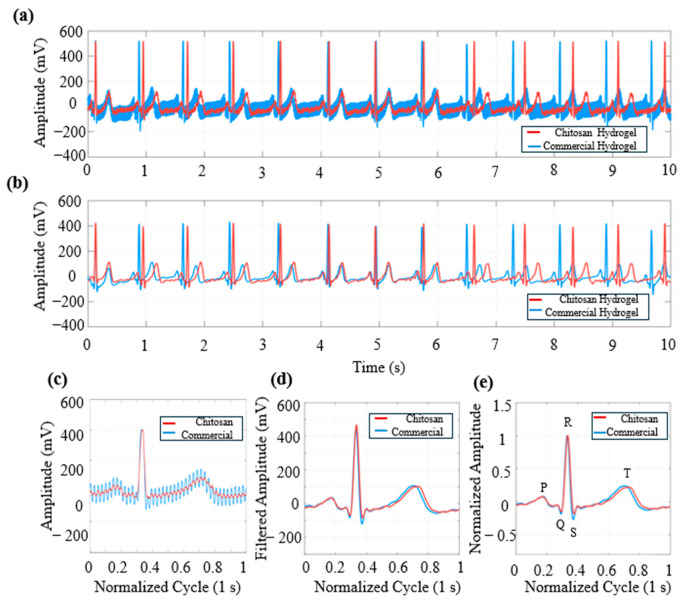
Temporal and morphological comparison of ECG signals acquired from chitosan-based hydrogel and commercial hydrogel. (**a**) Raw ECG signal acquired using the chitosan-based hydrogel electrode compared with a commercial hydrogel. (**b**) Filtered ECG signal acquired using the chitosan-based hydrogel electrode compared with a commercial hydrogel. (**c**) Raw signal from a single cardiac cycle, acquired from chitosan hydrogel and commercial hydrogel. (**d**) Filtered signal from a single cardiac cycle, acquired from chitosan hydrogel and commercial hydrogel (**e**) Normalized signal from a single cardiac cycle, acquired from chitosan-based hydrogel and commercial hydrogel.

Electrocardiogram (ECG) signals were acquired using a chitosan-based hydrogel and a commercial hydrogel, both interfaced with the same commercial Ag/AgCl sensing electrode, to evaluate the ability of the hydrogels to mediate physiological signal transmission at the skin–electrode interface. The resulting ECG recordings are shown in Figure 7. Raw ECG signals recorded under both hydrogel conditions (Figure 7a) exhibited recurrent cardiac waveforms throughout the acquisition period, with comparable waveform morphology across successive cardiac cycles. After signal filtering (Figure 7b), the ECG recordings obtained using the chitosan-based hydrogel and the commercial hydrogel preserved comparable temporal profiles, whit identifiable waveform features associated with the cardiac cycle. To further assess signal morphology, individual cardiac cycles were extracted from the raw recordings, and time-normalized (Figure 7c,d). The extracted cycles obtained from both hydrogels displayed similar waveform shape and temporal alignment. Normalized ECG waveform (Figure 7e) revealed consistent morphological characteristics (P, Q-R-S and T wave) between the chitosan-based hydrogel and the commercial hydrogel, including the main deflection of the cardiac cycle. These results indicate that the ECG waveform morphology obtained using the chitosan-based hydrogel is comparable to that obtained using the commercial hydrogel under the same measurement conditions.

#### 3.4.2. ECG Spectral Characteristics and Signal Metrics

**Figure 8:**
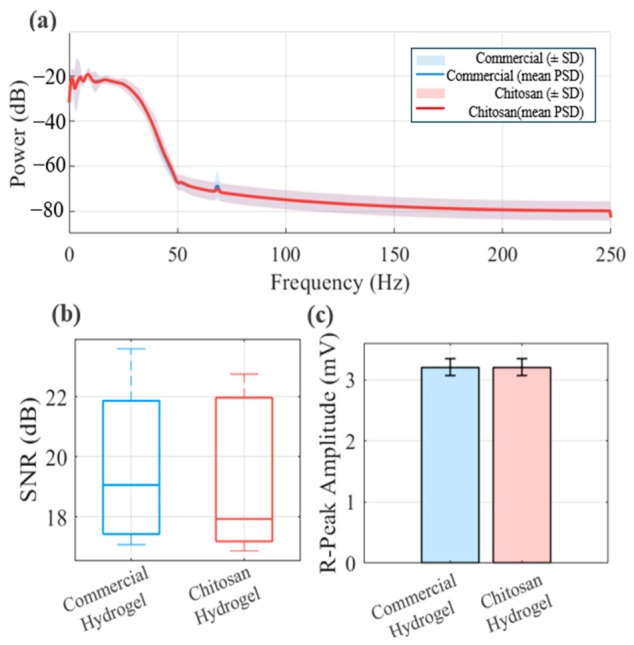
Comparison of ECG spectral characteristics and signal quality between a commercial hydrogel and a chitosan-based hydrogel. (**a**) Power spectral density (PSD) of ECG signals recorded with the commercial electrode and a chitosan-based hydrogel. Solid lines represent the mean PSD, while shaded regions indicate ±1 standard deviation across measurements over the analyzed frequency range. (**b**) Signal-to-noise ratio (SNR) of ECG recordings obtained with the commercial electrode and the chitosan-based hydrogel. (**c**) R-peak amplitude of ECG signals recorded with the commercial electrode and the chitosan-based hydrogel, reported as mean ± standard deviation.

The spectral characteristics and signal metrics of the ECG recordings obtained using the commercial hydrogel and the chitosan-based hydrogel are shown in Figure 8. The power spectral density (PDS) of the ECG signals (Figure 8a) exhibited comparable spectral distributions for both hydrogels across the analyzed frequency range, with similar attenuation profiles at higher frequencies. The signal-to-noise ratio (SNR) values extracted from the ECG recordings (Figure 8b) showed overlapping distributions between the commercial hydrogel and the chitosan-based hydrogel, indicating comparable signal quality between both hydrogel interfaces. In addition, the R-peak amplitude measured from the ECG signals (Figure 8c) presented similar mean values for both hydrogels, with no marked differences in peak magnitude. Together, these results indicate that the chitosan-based hydrogel ECG signal acquisition is consistent with spectral characteristics and signal metrics comparable to those obtained using the commercial hydrogel interface.

**Figure 9:**
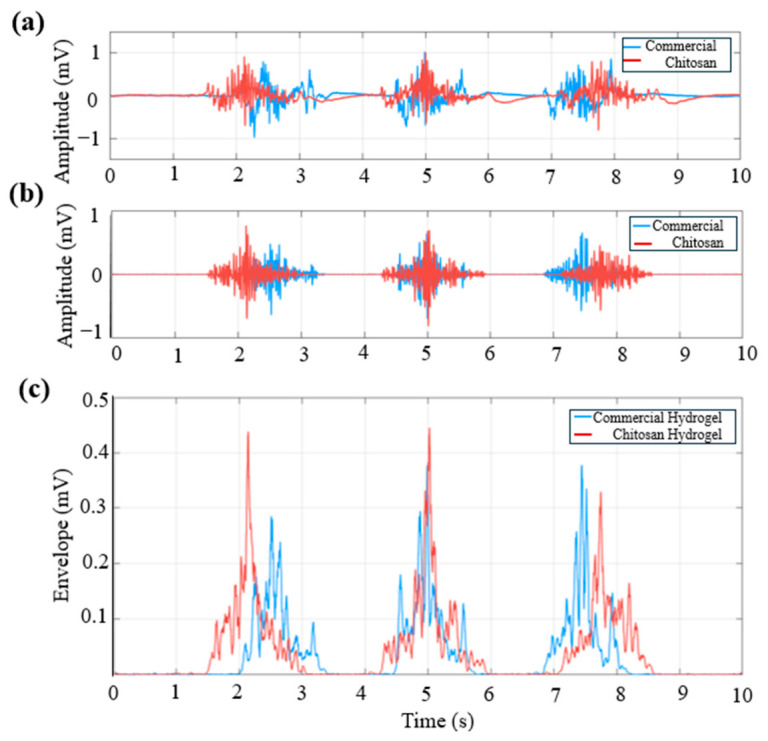
Representative EMG signal acquisition and processing using a commercial hydrogel and a chitosan-based hydrogel. (**a**) Raw EMG recorded whit using a commercial hydrogel and a chitosan-based hydrogel. (**b**) Filtered EMG signals obtained from the previous raw signals recordings. (**c**) EMG signal envelope extracted from the filtered signals.

Electromyography (EMG) signals were recorded using the same Ag/AgCl electrode eighter with a commercial hydrogel or with the chitosan-based hydrogel, and the resulting signals are presented in Figure 9. Raw EMG recordings obtained using both hydrogel interfaces (Figure 9a), exhibited clear periods of muscle activation and relaxation, with clearly distinguishable periods of muscle activation and relaxation throughout the acquisition window. After band-pass filter (Figure 9b), the EMG signals obtained with the commercial hydrogel and those obtained with the chitosan-based hydrogel showed a marked reduction in baseline noise while preserving the temporal structure of muscle activation bursts. The filtered signals displayed comparable activation timing and waveform profiles across the analyzed contraction periods.

To further characterize muscle activation, the EMG signal envelope was extracted from the filtered recordings (Figure 9c). The resulting envelopes highlighted the temporal modulation of muscle activity during successive contractions, with comparable activation patterns observed for recording acquired using the same Ag/AgCl sensing electrode, with the skin–electrode interface mediated by eighter the commercial hydrogel or the chitosan-based hydrogel.

#### 3.4.3. EMG Spectral Characteristics and Signal Metrics

**Figure 10:**
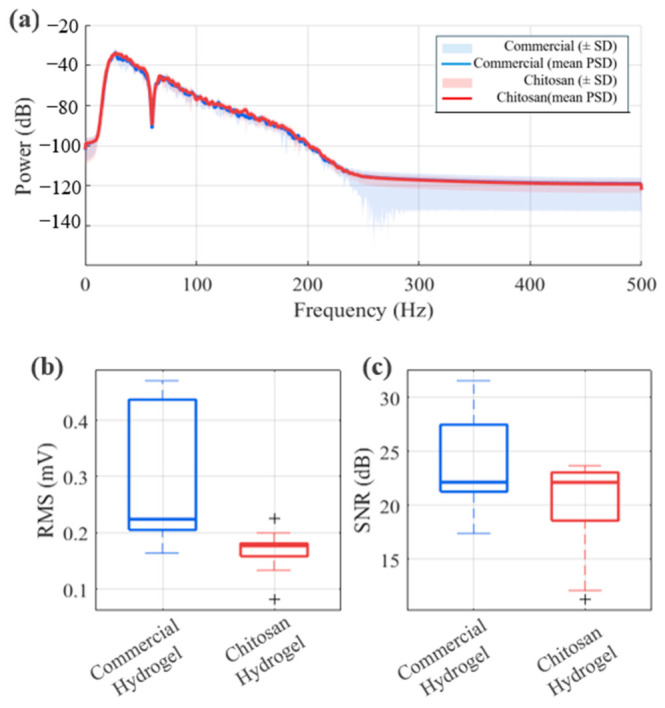
Comparison of EMG spectral characteristics and signal quality between a commercial hydrogel and a chitosan-based hydrogel. (**a**) Power spectral density (PSD) of EMG signals recorded with the commercial hydrogel and a chitosan-based hydrogel. Solid lines represent the mean PSD, while shaded regions indicate ±1 standard deviation across measurements over the analyzed frequency range. (**b**) Root mean square (RMS) amplitude of EMG signals recorded during muscle contraction using the commercial electrode and a chitosan-based hydrogel. (**c**) Signal-to-noise ratio (SNR) of EMG recordings obtained with the commercial electrode and a chitosan-based hydrogel. For the boxplots, boxes represent the interquartile range (IQR), the central line indicates the median, and “+” indicates outliers.

The spectral characteristics and signal metrics of the EMG recordings obtained using the commercial hydrogel interface and the chitosan-based hydrogel interface are presented in Figure 8. The power spectral density (PSD) of the EMG signals (Figure 10a) exhibited similar spectral profiles for both interfaces across the analyzed frequency range, with comparable distributions of spectral power concentrated at low mid frequencies.

Quantitative analysis of the EMG amplitude revealed differences in root mean square (RMS) values between the two hydrogels (Figure 10b), reflecting variations in signal amplitudes during muscle contraction. Despite these differences, the overall spectral content remained comparable. The signal-to-noise ratio (SNR) values derived from the EMG recordings (Figure 10c) showed overlapping ranges between the commercial hydrogel and the chitosan-based hydrogel interfaces, indicating comparable signal quality for EMG acquisition.

## 4. Discussion

### 4.1. Material Characterization of the Extracted Chitosan

The present study focused on a proof-of-concept functional evaluation of chitosan-based conductive hydrogel as an interface material for physiological signal acquisition.

Figure 4 presents the material characterization of the extracted chitosan through FTIR analysis and static light scattering measurements. The FTIR spectrum exhibited the characteristic absorption bands commonly associated with chitosan-based materials, including the broad O–H/N–H stretching region and the amide-associated absorption bands. The relative intensity of the amide-related bands suggested that the extracted material was predominantly deacetylated, consistent with the expected characteristics of chitosan obtained through alkaline deacetylation processes.

The molecular weight estimated from static light scattering analysis was $M_{w}=4.63\times 10^{4}g/mol$, as determined from the intercept of the Debye plot shown in Figure 4b. The linear relationship observed throughout the analyzed concentration range $\left(R^{2}=0.9954\right)$ indicated stable scattering behavior under the evaluated experimental conditions. Both molecular weight and degree of deacetylation are known to influence physicochemical properties such as polymer chain interaction, viscosity, hydrogel formation, and ionic transport behavior in chitosan-based systems. In the present study, these measurements provide additional material characterization context for interpreting the mechanical and electrical behavior of the developed hydrogel interface.

### 4.2. Mechanical Behavior of the Hydrogel Interface

Figure 5 shows the mechanical response of the chitosan-based hydrogel obtained from a uniaxial compression test performed on a small square hydrogel sample. Compressive stress increases nonlinearly with increasing strain: at low strain the stress rises gradually whereas at higher strain the curve becomes progressively steeper, indicating increased resistance to further compression within the tested range. This nonlinear compressive response is consistent with the behavior commonly reported for hydrated polymer networks, where the apparent stiffness increases as compression progresses [55,56].

Similar nonlinear compressive responses have been reported for chitosan/PVA composite hydrogels prepared via physical crosslinking methods, where low initial stiffness combined with strain-dependent stiffening is commonly observed. Reported mechanical responses for chitosan/PVA systems vary widely depending on polymer ratio, crosslinking strategy, and testing protocol, which limits direct quantitative comparison. Within this context, the compressive behavior observed in the present study is consistent with previously reported chitosan/PVA composite gels intended for compliant interface applications rather than load bearing uses.

The observed compressive compliance is consistent with previous reports describing compliant hydrogel interfaces under compressive loading conditions [57,58]. These implications are discussed qualitatively based on the compression curve, without additional mechanical modeling.

Regarding the tensile test, our chitosan hydrogel exhibited a Young’s modulus of 0.695 MPa, an ultimate tensile strength of 0.332 MPa, and an elongation at break of 111.87%. Tensile characterization is commonly reported for physically crosslinked chitosan/PVA hydrogel systems intended for flexible biomedical applications, where the ability to undergo deformation under tensile loading is considered an important mechanical characteristic [59,60,61]. In the present study, the hydrogel remained deformable under tensile loading and exhibited an elongation at break exceeding 100%. Furthermore, the tensile stress–strain response exhibited multiple stress-drop events prior to complete fracture. As shown in Figure 5f–i, these events coincided with visible changes observed in the hydrogel film during tensile loading.

In addition to compressive compliance, the chitosan-based hydrogel exhibited resistance during unloading following compressive deformation, as observed in the loading–unloading response shown in Figure 5. This behavior is consistent with the viscoelastic characteristics commonly observed in physically crosslinked polymeric hydrogel systems [62,63]. In the present study, this resistance force is reported as an interface-level observation rather than a quantified adhesive property, consistent with its intended role in short-term applications.

### 4.3. Electrical Characterization of the Chitosan-Based Hydrogel

The electrical behavior of the chitosan-based hydrogel was evaluated in terms of its role as an interface between the skin and a commercial Ag/AgCl sensing electrode. As shown by the impedance spectroscopy results revealed that both hydrogels (chitosan-based and commercial ones) exhibited frequency-dependent impedance magnitude and phase response typical of ionic hydrogel interfaces [64]. The overall impedance trends observed for the chitosan-based hydrogel were comparable to those obtained using the commercial hydrogel across the analyzed frequency range.

Impedance spectroscopy results (Figure 6) show that the chitosan-based hydrogel and the commercial hydrogel present distinct impedance magnitude and phase values across the analyzed frequency range. The chitosan-based hydrogel exhibited higher impedance magnitude compared to the commercial hydrogel, particularly at higher frequencies, indicating differences in the electrical response of the two hydrogel interfaces.

Despite these differences, both hydrogels’ interfaces displayed smooth and continuous frequency-dependent impedance magnitude and phase responses, without abrupt transitions or irregular behavior [65]. This indicates coherent electrical behavior between the sensing electrode and both hydrogel interfaces. The phase response followed a gradual frequency-dependent trend in both cases, indicating continuous and frequency-dependent electrical behavior across the analyzed range. Overall, these results indicate that although the impedance profiles differ between the two hydrogels, both supported comparable electrical response behavior under the tested conditions.

Although no direct microstructural characterization of the hydrogel network (e.g., SEM or porosity analysis) was performed, the observed impedance behavior is consistent with that reported for hydrated polymer networks acting as ionic conduction pathways in skin–electrode interfaces. Recent studies have shown that continuous, physically crosslinked hydrogel matrices can provide stable frequency-dependent impedance responses suitable for electrophysiological sensing, even in the absence of detailed structural imaging, when the focus is placed on functional interface performance rather than material morphology [66,67,68]. In this context, the present impedance results support the feasibility of the chitosan-based hydrogel as a functional electrical interface material.

### 4.4. Interpretation of ECG Signal Morphology and Spectral Characteristics

The ECG results describe signal morphology and frequency-domain characteristic obtained using a chitosan-based hydrogel in comparison with a commercial hydrogel, both coupled to the same Ag/AgCl sensing electrode. As shown in Figure 7a, ECG waveforms acquired using the chitosan-based hydrogel, exhibit clearly defined cardiac features that are comparable to those obtained using the commercial hydrogel. Following filtering and cycle normalization, the main morphological components of the ECG signals, including the P wave, QRS complex and T wave remain clearly identifiable in recordings obtained with both hydrogels.

The extracted and normalized cardiac cycles show similar waveform shapes and preserved sequence of the main ECG deflections, suggesting that the chitosan-based hydrogel does not introduce observable changes in ECG waveform morphology relative to the commercial hydrogel under the tested conditions. The results obtained in the frequency domain show similar trends to those observed in the time domain. As shown in Figure 5a, the power spectral density of the ECG signal recorded using both hydrogels shows overall comparable spectral shape across the analyzed frequency range.

Quantitative signal metrics, including the signal-to-noise ratio and R-peak amplitude (Figure 7b,c), are within comparable range for both hydrogels, with overlapping distributions and similar mean values. Taken together, the ECG waveforms and PSD profiles obtained using the chitosan-based hydrogel appear comparable to those recorded with the commercial hydrogel when coupled to the same Ag/AgCl electrode, with no visually apparent differences in waveform structure or overall frequency content.

These observations are aligned with recent reports on hydrogel-based skin interfaces, where hydrated and compliant ionic materials have been shown to preserve ECG morphology and spectral content by enabling conformal contact and stable ionic coupling at the skin–electrode interface [69,70] from an applied perspective, the present results indicate that the chitosan-based hydrogel functions as a passive interface layer that does not distort low-frequency biopotential components relevant for ECG acquisition.

### 4.5. EMG Signal Characteristics: Amplitude Differences and Spectral Density

Figure 8 summarizes EMG acquisition using a chitosan-based hydrogel and a commercial hydrogel, each using the same Ag/AgCl electrode type. Across the 10 s segments shown, each recording exhibits repeated activation burst separated by baseline-level intervals; within each trace, three prominent bursts are observable, indicating a qualitatively similar burst activation-rest pattern across the two electrode interfaces. Filtering (Figure 8b) improves the visual separation between the baseline and the activation periods within each recording, making burst boundaries more evident. The envelope representation (Figure 8c) highlights change in activation magnitude across the bursts within each recording. Although the recordings were acquired separately, they were obtained under the same experimental conditions and during voluntary contractions without external load. Within this context, the displayed segments show differences in envelope peak amplitude and intra-burst envelope shape between the chitosan-based hydrogel and the commercial hydrogel recordings. These differences are reported as observable features of the presented traces and are not attributed to a specific underlying mechanism.

Figure 9 compares EMG spectral characteristics and signal quality between recordings obtained using the chitosan-based hydrogel and the commercial hydrogel. In the frequency domain (Figure 9a), both conditions show a very similar overall PSD profile across the analyzed frequency range, and the variability bands (±SD) largely overlap, consistent with comparable spectral distributions.

Amplitude-related differences are evident in Figure 9b. The RMS amplitude distribution for the commercial electrode is shifted toward higher values compared to the chitosan-based hydrogel, which shows a more compact distribution at lower RMS levels in the displayed dataset. Similarly, the SNR distributions (Figure 9c) overlap between the two interfaces; however, the commercial hydrogel shows higher central values, whereas the chitosan-based hydrogel exhibits a wider spread that includes lower SNR values.

Taken together, the PSD profiles appear comparable, whereas the quantitative metrics shown in Figure 9 (RMS and SNR) differ between the two EMG hydrogel interfaces in the presented recordings. These differences are reported descriptively from the displayed data and are not attributed to a specific cause.

EMG interfaces, where interface-dependent variations in amplitude-related metrics were observed despite similar spectral characteristics. Such differences are commonly attributed to contact conditions, noting that EMG amplitude metrics are particularly sensitive to interface impedance and conformability rather than changes in muscle activation itself [68,71]. These differences may reflect interface-related variations rather than intrinsic limitations of the hydrogel material itself. Comparable observations have been reported in recent studies on hydrogel-based.

### 4.6. Limitations and Future Work

This study has some limitations that should be considered. First, the hydrogel network was strengthened using physical crosslinking only. While the compressive response was consistent with values reported for similar chitosan-based hydrogels, excessive localized pressure applied at the lead-pin/electrode connection interface could produce visible damage to the hydrogel structure during handling. This effect was associated with stress concentration at the contact point rather than with the overall compressive behavior of the material.

Future work will aim to improve the mechanical durability of under localized loading by implementing chemical crosslinking strategies and/or introducing a protective layer to distribute the pressure at the cable–electrode contact point. While the present dataset supports the comparison reported here, additional repeated trials may be conducted to quantify within-subject variability. Finally, future studies may evaluate signal behavior under motion and environmental variation conditions.

From a practical standpoint, the chitosan-based hydrogel was treated as a disposable or limited-use interface layer. This usage model is consistent with current trends in hydrogel-based electrophysiological interfaces, where disposability is often favored due to dehydration, hygiene considerations, and performance drift over time [72,73]. Reuse and long-term stability were not evaluated and remain outside the scope of the present study. As a proof-of-concept interface study, the present work prioritized preliminary functional signal acquisition.

## 5. Conclusions

This study evaluated a chitosan-based hydrogel as an electrode interface for physiological signal acquisition and compared it with a commercial hydrogel under comparable experimental conditions. For ECG, the chitosan-based hydrogel produced recordings in which the main waveform features were identifiable, and the frequency-domain results showed comparable PSD profiles and R-peak amplitudes within the reported variability. For EMG, the PSD profiles were also comparable between interfaces, whereas RMS amplitude and SNR differed across the presented recordings.

Mechanical testing using uniaxial compression showed a nonlinear compressive response, with higher resistance at large compressive strain. Overall, the results presented here show that the chitosan-based hydrogel can be used to acquire ECG and EMG signals under the tested experimental conditions. These findings support the feasibility of the proposed hydrogel as a proof-of-concept conductive interface for physiological signal acquisition.

## Figures and Tables

**Table 1:** Representative hydrogel formulations evaluated during preliminary formulation screening.

Formulation	Chitosan (4%)	Glycerol	PVA	NaCl	Film Formation
F1	5 mL	1 mL (10%)	3 mL (10%)	0.1 g	No
F2	5 mL	2 mL (15%)	0.5 mL (10%)	0.1 g	No
F3	5 mL	0.5 mL (10%)	2 mL (15%)	0.1 g	Partial
F4	5 mL	0.5 mL (10%)	2 mL (10%)	0.2 g	Partial
F5	5 mL	0.5 mL (10%)	2 mL (10%)	0.3 g	No
Final Formulation	5 mL	0.5 mL (10%)	2 mL (10%)	0.1 g	Yes

Note: Film formation classification: Yes = continuous hydrogel film obtained; Partial = hydrogel film obtained with incomplete consolidation or poor cohesion; No = continuous hydrogel film not obtained, resulting in a liquid or non-self-supporting material.

## Data Availability

The original contributions presented in this study are included in this article; further inquiries can be directed to the corresponding authors.
